# Effectiveness of the acceptance and commitment therapy for resilience promotion in a non-clinical sample: A randomized controlled and a pilot study

**DOI:** 10.1186/s40359-025-03023-1

**Published:** 2025-07-01

**Authors:** Engin Büyüköksüz

**Affiliations:** Psychological Counseling and Guidance Center, Istanbul Technical University, Istanbul, Turkey

**Keywords:** Acceptance and commitment therapy, Resilience, Randomized controlled, Intervention, Mediation analysis

## Abstract

**Background:**

This research investigates the efficacy of Acceptance and Commitment Therapy (ACT) in fostering resilience among non-clinical adolescents. The prevailing socio-economic and psychological challenges have notably affected vulnerable demographics, particularly adolescents, thereby underscoring the importance of resilience for mental health.

**Methods:**

This randomized controlled pilot study, carried out in Istanbul, sought to assess the effects of ACT interventions on resilience, self-compassion, and psychological flexibility in high school students. A total of 89 participants were assigned to either the ACT intervention group or a control group, with the intervention comprising six weekly sessions that emphasized key ACT principles, including mindfulness, emotional regulation, and self-compassion.

**Results:**

Utilizing the Connor-Davidson Resilience Scale, Self-Compassion Scale, and Avoidance and Fusion Questionnaire, the findings indicated significant enhancements in resilience and self-compassion, alongside a decrease in psychological inflexibility within the ACT intervention group compared to the control group.

**Conclusion:**

This pilot study provides preliminary evidence that ACT may be a promising approach for enhancing psychological resilience and self-compassion among adolescents. However, given the small sample size and baseline differences between groups, these findings should be interpreted with caution and warrant further investigation with larger, more diverse populations.

## Introduction

In the current era resilience and resiliency have garnered significant attention for many reasons. The global landscape is currently facing crises on multiple interconnected fronts, including political, social, economic, and environmental challenges. These crises particularly affect women, low socioeconomic status individuals, and children.

Adolescence is a critical developmental period characterized by physical, cognitive, and psychosocial development from childhood to adulthood and leads to important changes (World Health Organization [WHO]) [1]. By the time individuals reach the age of 14, around half of all mental health disorders that will manifest in adulthood have already started to develop [2]. Unfortunately, the majority of these cases remain undetected and untreated. The majority of mental disorders typically emerge between the ages of 12 and 25, impacting approximately 20% of adolescents [3, 4]. The challenges faced during adolescence are frequently rooted in the concerns surrounding self-evaluation. This period, which can be particularly complex for adolescents, may disrupt the healthy development process of young people and may also make them vulnerable to psychological problems. Negative adolescent experiences can have lasting consequences on future well-being.

According to Beauvais and Oetting [5], it has been stated that certain factors contribute to fostering positive outcomes in young individuals, helping them mitigate the effects of risks. These factors can be categorized as either resources or assets. Resources are external factors that aid young individuals in overcoming risks. Supportive resources may encompass parental involvement, mentorship from adults, or community organizations dedicated to fostering the positive growth of youth individuals [6]. On the other hand, Assets refer to the positive attributes that exist within the individual, such as their competence [6], mindfulness [7, 8], self-compassion [9, 10], and self-efficacy [11, 12], self-esteem [13, 14], and psychological flexibility [15–17].

Skills such as resilience, self-esteem, and self-efficacy can protect adolescents’ mental health [18–20]. Prevention intervention can protect adolescents from events that have not yet occurred [21]. Positive psychological experiences can help protect the mental health of adolescents by increasing their resilience [2]. The concept of resilience, defined as the capacity to navigate and flourish despite challenges or adversity, is pertinent across all phases of development [22, 23]. Resilience is recognized as the capacity to sustain or regain positive adaptation, mental well-being, or psychosocial functioning in the face of significant challenges [24]. Interventions that enhance mindfulness and nonjudgmental acceptance of the present circumstances, particularly during difficult times, have been shown to enhance resilience [25, 26].

Resilience is understood as a dynamic process characterized by the interplay of risk and protective factors, which can be both internal and external to the individual, this interaction serves to influence the impact of negative life events [27]. Identifying protective factors in children and adolescents as they navigate challenges is fundamental to resilience-focused interventions [28, 29].

Given the complex nature of resilience and the various protective factors that contribute to its development, has resulted in the development of a range of psychoeducational programs of varying lengths designed to bolster resilience in different demographic groups [30]. Research indicates that interventions combining cognitive-behavioral therapy (CBT) with additional approaches, such as mindfulness practices, have been effective in enhancing resilience [31) in both the general population [32] and among adolescents [33].

Techniques derived from acceptance and commitment therapy (ACT) that focus on enhancing psychological flexibility promote a transformation in how clients engage with their emotions and thoughts through the cultivation of mindfulness, which is characterized by a nonjudgmental awareness of the present moment. The components of the ACT framework on psychological well-being are discussed in scholarly literature, particularly with the concept of self-compassion [34, 35]. Furthermore, certain processes integral to ACT have been associated with self-compassion [36, 37].

### Purpose of this study

Enhancing effective intervention strategies can lead to improved developmental outcomes during adolescence and facilitate the implementation of scientifically testable resilience interventions. Studies on adolescent resilience in the literature focus on protective processes at three levels: individual, family, and community. This study includes protective processes at the individual level: (1) To show adolescents that they have the resources within themselves to successfully adapt to the changing physical, psychological, and social environment. (2) Ensuring that adolescents develop the capacity to adapt constructively to adversities, thereby fostering resilience. (3) Supporting adolescent mental health through resilience interventions implemented in schools, particularly for vulnerable and disadvantaged groups.

In summary, there is a significant increase in the risk of psychological and behavioral problems during adolescence. Positive psychological experiences can help protect mental health by increasing resilience. ACT can support the mental health of adolescents through a set of treatments. The first aim of this study is to examine the effectiveness of acceptance and commitment therapy in promoting resilience within a non-clinical sample. The second aim is to explore whether psychological flexibility (as an ACT intervention) predicts resilience through self-compassion.

### Method

This study is randomized controlled and to evaluate the effectiveness of ACT for resilience promotion in a non-clinical sample. This study was conducted in a high school in Istanbul. Primary outcomes were assessed at baseline, immediately post-intervention. This study was performed in line with the principles of the Declaration of Helsinki. Ethical approval was obtained from the Ethics Committee of Istanbul Technical University (Date: 06/12/2023; Protocol number: 431), and the Istanbul Provincial Directorate of National Education Research Ethics Committee (Approval number: E59090411-20-98649264). Informed consent was obtained from all individual participants included in the study.

### Participants

Participants were recruited from the Istanbul Kadikoy High School through posters and announcements. The population was non-clinical adolescents who wanted to promote resilience. The inclusion criteria were as follows: (a) Age between 16 and 18 years; (b) enrollment as a student at Istanbul Kadikoy High School; (c) self-reported psychological and physical wellness (non-clinical) (d) willingness to attend group-based resilience training; (e) parental informed consent and student assent. We also clarified how willingness to “promote resilience” was operationalized—through voluntary attendance at the program introduction session and expression of interest. These clarifications ensure transparency and replicability, in alignment with the study’s purpose and target population.

Individuals were excluded from the study if they met any of the following criteria: (a) did not express interest in participating in group-based skills training; (b) lacked parental consent for participation in group-based skills training; (c) had a diagnosed psychiatric disorder or special educational needs; (d) had experienced the earthquake in Türkiye in February 2023; or (e) were currently receiving other forms of psychotherapy. Participants who had experienced the 2023 earthquake were excluded due to the potential psychological impact of acute trauma. Such experiences may have independently influenced the variables of interest—particularly resilience and psychological flexibility—and could have confounded the effects of the intervention.

Of the 205 students that met baseline inclusion criteria 89 agreed to participate in the study. Participants were assigned randomized to groups (ACT intervention [ACTi] or control group). Four individuals from this group dropped out after randomization and therefore were not included in further analyses. Five individuals who did not attend two of the six sessions were not included in the analysis. Women constituted the larger portion of the remaining sample of 56 (70%). The age of the participant’s mean was 16.95 years (SD = 0.35; range = 16–18).

### Procedure

**Fig. 1:**
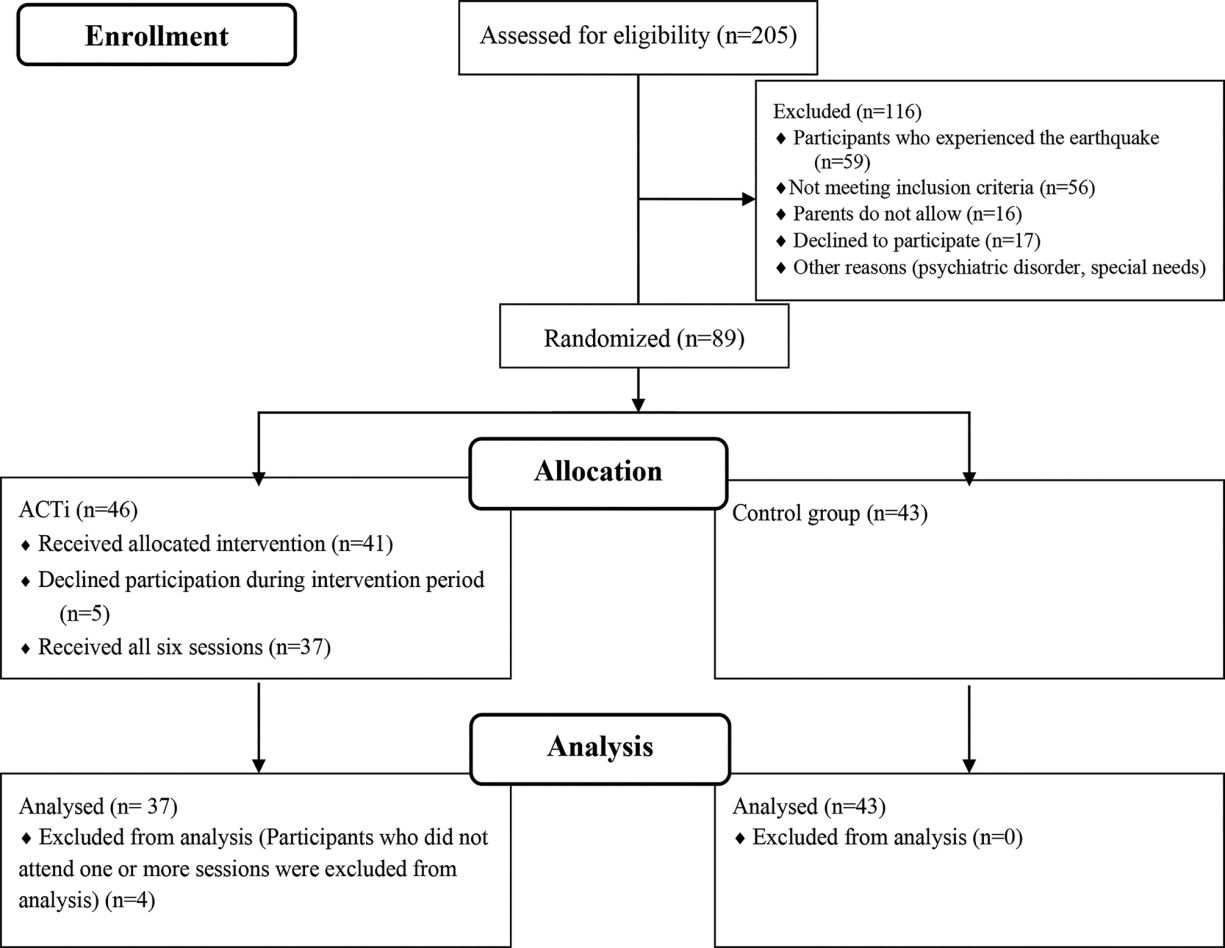
Participant flow diagram based on CONSORT 2010 guidelines. **Note**: ACTi: ACT intervention

The participants and their parents were announced detailed information on the study, in/exclusion criteria. Participants and their parents obtained informed consent. All personal identifiers were eliminated during the data processing phase, and the data was securely stored on encrypted servers with access limited to authorized researcher. Furthermore, those managing the data signed confidentiality agreements, and the study complied rigorously with the approved ethical protocol. The study was conducted using a self-report scale. Upon the completion of the baseline questionnaire, the participants were randomly allocated to either the ACTi or the control group. To allocate participants to specific conditions, the researcher employed a random selection method by drawing a slip of paper from an envelope that originally held 89 slips, with 43 designated for each condition. After each selection, the drawn slip was removed to maintain a balanced distribution of participants across groups over time. However, as the number of registered slips diminished, a coin flip was utilized to assign the last three participants to their respective groups, compensating for the altered probabilities of selection. Consequently, the ACTi group ultimately comprised 46 participants. Participants in the experimental group received six intervention sessions (80–90 min) once in week. No program was applied to the control group. The post-intervention was conducted, after the sixth-intervention session assessment. After completing the sixth intervention session, ACTi participants received a cinema ticket as compensation. The control group was not compensated. During the intervention period, five participants declined participation due to reasons such as scheduling conflicts, lack of interest, or personal circumstances. Additionally, four participants were excluded from the analysis as they did not attend one or more intervention sessions. The primary reasons for missing sessions included illness (e.g., influenza) and school absence (Fig. 1.).

## Intervention

The ACTi, “Promotion Resilience” was designed for adolescents. The development process was described in detail in this article. The program was in-person and contained six sessions. The intervention program was developed through ACT literature [36, 38, 39]. The six sessions were organized in a predetermined sequence, occurring weekly. Although self-compassion is not a traditional core process within ACT, it was purposefully included in this intervention due to its conceptual alignment with ACT’s theoretical underpinnings. Both ACT and self-compassion emphasize processes such as defusion, self-as-context, and values-based action, all of which contribute to psychological flexibility. According to Yadavaia, Hayes, and Vilardaga [35], ACT can be used to enhance self-compassion, and vice versa, as these frameworks share mutually reinforcing mechanisms. In the context of adolescent resilience, fostering self-compassion supports acceptance and reduces harsh self-judgment, which are key therapeutic goals in ACT. Therefore, self-compassion was integrated as a complementary process to strengthen the effectiveness of the intervention. Each module was designed to take approximately 80 to 90 min to complete. The sessions were structured around the six fundamental processes of ACT, known as “Hexaflex” which aim to improve psychological flexibility [36]. These concise components were consistent with the key processes highlighted in ACT. Within the framework of ACT, mindfulness strategies are employed to help individuals enhance their awareness of thoughts and emotions, thereby fostering increased psychological flexibility [36] and resilience [40]. The “Promotion Resilience” component did not include any homework assignments and consisted entirely of experiential sessions (Table 1).

**Table 1:** Content of sessions

Session	Aim of Session	Techniques Used
Session 1	Application of warm-up and introduction exercises, explaining the psycho-educational program, group rules, personal goals and values, understanding resilience	Warm-up exercises, explanation of resilience, group rule setting, personal goal identification, mindfulness exercise
Session 2	Understanding feelings and emotions, learning how we regulate our emotions, using mindfulness and acceptance techniques, recognizing the link between emotion regulation and resilience	Mindfulness meditation, acceptance techniques, emotion regulation strategies (ice cube exercise)
Session 3	Understanding values and experiential avoidance, continuing in line with values during emotional challenges, using mindfulness and self-compassion exercises, recognizing the link between self-compassion and resilience	Mindfulness exercise, bus metaphor, self-compassion meditation, experiential avoidance awareness
Session 4	Recognizing social support resources, understanding values and social solidarity, using mindfulness and self-compassion for sustainable commitment	Mindfulness exercise, string game, social support identification
Session 5	Developing empathy through mindfulness, using self-as-context and self-compassion for resilience	Mindfulness exercise, eye contact exercise, empathy practices, self-compassion techniques, the ACT Matrix
Session 6	Wrapping up and farewell, practicing music mindfulness, sharing group experiences, reevaluating personal goals and values	Music mindfulness, group reflection, personal goal reevaluation

### Measurements

#### The 10-item connor-davidson resilience scale (10-item CD-RISC or CD-RISC-SF)

The 10-item Connor-Davidson Resilience Scale shortened by Campbell-Sills and Stein [41], was translated into Turkish by Kaya and Odacı [42]. It is a 10-item scale with a 5-point Likert rating (0, “not true at all”; 4, “true nearly all the time”) (e.g. “a*ble to adapt to change*”). The total score on the scale ranges from 0 to 40, with higher scores indicating a greater level of resilience. The CD-RISC-SF has a single-factor structure. The Cronbach’s alpha coefficient was found to be 0.81. In the current study, Cronbach’s alpha coefficient for the scale was determined to be 0.80 for baseline scores.

#### Self-compassion scale–short form (SCS–SF)

The SCS-SF, an English version of the Self-Compassion Scale originally developed by Raes et al. [43] and later translated into Turkish by Büyüköksüz [44], is a 12-item tool used to assess self-compassion. The fit indices of the six-factor model demonstrated satisfactory results, indicating that the model adequately represents the underlying structure of the scale. Each sub-dimension consists of two items, and respondents rate these items on a 5-point Likert-type scale ranging from 1 (“almost never”) to 5 (“almost always”) (e.g. “w*hen I fail at something important to me I become consumed by feelings of inadequacy*”). The total score on the scale ranges from 12 to 60, with higher scores indicating a greater level of self-compassion. The internal reliability coefficient of the scale, as measured by Cronbach’s alpha, was found as 0.86 in the original study by Raes et al. [43]. In the Turkish study, Cronbach’s alpha coefficient of the scale was found as 0.80 [44]. In the current study, Cronbach’s alpha coefficient for the scale was determined to be 0.78 for baseline scores.

#### Avoidance and fusion questionnaire for youth 8 (AFQ-Y8)

The English version of the AFQ-Y8, developed by Greco et al. [45] and translated into Turkish by Büyüköksüz [44], consists of 8 items that assess levels of experiential avoidance and cognitive fusion. Respondents rate each item on a 5-point Likert-type scale, ranging from 0 = “not at all true” to 4 = “*very true*” (e.g. “*I am afraid of my feelings*”). The total score ranges from 0 to 32, with higher scores indicating greater levels of experiential avoidance and cognitive fusion. The AFQ-Y8 demonstrates acceptable internal consistency reliability, with a Cronbach’s alpha coefficient of 0.90. This scale measures characteristics related to the experiential avoidance and cognitive fusion subscales within psychological inflexibility as proposed by Greco et al. [45], as part of ACT. In the Turkish study, Cronbach’s alpha coefficient was reported as 0.84 (*n* = 467) and 0.87 (*n* = 405) [46]. In the present study, Cronbach’s alpha coefficient for the scale was calculated as 0.75 for baseline scores.

### Analyses

All analyses were conducted with IBM SPSS Statistics^®^ 28. Skewness and kurtosis assessments were used to test normality assumptions [47]. Table 2 presents descriptive statistics and normality assumptions, including baseline and post-intervention measurements for the ACTi and Control Group. First, an independent samples t-test was conducted to explore differences in baseline scores between the ACTi and the control group. Second, post-intervention scores between the ACTi and control group were compared through an independent samples t-test. Third, the effectiveness of the intervention was assessed by conducting repeated measures analyses on baseline and post-intervention outcomes, with group and time (baseline and post-intervention) being considered.Effect sizes were calculated using Cohen’s d values for t-tests, categorized as small (0.20–0.49), medium (0.50–0.79), and large (0.80–1.00) [48].

Finally, mediation analysis was conducted using the SPSS PROCESS Macro. The primary conjectures of this investigation were based on the work of Hayes and Rockwood [49]. To assess the sample distribution for the mediation analysis, the Bootstrap confidence interval (CI) technique proposed by Shrout and Bolger [50] and further refined by Preacher and Hayes (2004, 2008) [51, 52] was employed. The mediation effect’s Bootstrap resampling was performed 5,000 times and computed using the Bias-Corrected (BC) Bootstrapped CI (95% CI) [49].

In the mediation analyses, the ACTi and control group were defined as the independent variable, while post-intervention resilience scores served as the dependent variable. Post-intervention scores of self-compassion and psychological inflexibility (AFQ-Y8) were included as mediating variables to examine the indirect effects of the intervention. To ensure a more accurate estimation of the mediation pathways and to control for pre-existing differences between groups, baseline scores of self-compassion and psychological inflexibility were entered into the model as covariates. This approach allowed the analyses to isolate the effect of the intervention from participants' initial levels on these variables, thereby providing a more robust interpretation of the changes observed post-intervention and the mechanisms through which the intervention exerted its effects on resilience. 

## Results

A total of 205 individuals took part in the baseline survey (Fig. 1). Consequently, the final sample comprised 89 participants, who were randomly allocated to the ACTi group (*n* = 37) and the control group (*n* = 43). The descriptive statistics for both baseline and post-intervention measurements of the ACTi and control groups indicate significant differences in outcomes across the three scales (CDRS-SF, SCS-SF, and AFQ-Y8), underscoring the impact of the ACT intervention on depression, self-compassion, and anxiety, as presented in Table 2. Sociodemographic characteristics and other relevant variables are presented in Table 3. The two groups exhibited a significant difference in age (t (78) = -2.046, *p* <.05), while no significant difference was observed in terms of sex (χ2(1) = 1.056, *p* >.05). 

The ACTi and control group compared baseline scores of the CD-RISC-SF, the SCS-SF, and the AFQ-Y8 in Table 3. The participants in ACTi and the control group did not differ in CD-RISC-SF (t (78) -1.000, *p* >.05 d= -0.224). The participants in ACTi and control group did differ in the SCS-SF (t (78) -3.790, *p* <.001, Cohen’s d= -0.850), and the AFQ-Y8 (t (78) 5.338, *p* <.001, d = 1.197). The SCS-SF scores of the control group were higher than ACTi. The AFQ-Y8 scores of the control group were lower than ACTi.

**Table 2:** Descriptive statistics of the baseline and the post-intervention measurement at the acti and the control group

			Baseline						Post-intervention					
Group	Scale	*n*	Min	Max	Mean	SD	Kurtosis	Skewness	Min	Max	Mean	SD	Kurtosis	Skewness
ACTi	CDRS-SF	37	3	33	20.11	6.354	0.246	− 0.100	7	35	24.00	7.299	− 0.489	− 0.460
	SCS-SF	37	12	39	28.189	5.631	0.667	− 0.360	29	43	35.568	3.731	− 0.788	− 0.200
	AFQ-Y8	37	16	32	21.678	4.096	− 0.126	0.739	11	28	18.460	5.064	-1.159	0.303
Control Group	CDRS-SF	43	5	34	21.56	6.566	0.112	− 0.634	6	29	20.91	5.273	0.715	− 0.890
	SCS-SF	43	16	53	34.442	8.564	− 0.124	− 0.319	16	47	33.116	6.939	0.057	− 0.470
	AFQ-Y8	43	0	32	14.930	6.678	0.079	0.165	10	29	15.791	4.591	1.490	1.386

Note: ACTi: ACT intervention

The ACTi and control group compared baseline scores of the CD-RISC-SF, the SCS-SF, and the AFQ-Y8 in Table 3. The participants in ACTi and the control group did not differ in CD-RISC-SF (t (78) -1.000, *p* >.05 d= -0.224). The participants in ACTi and control group did differ in the SCS-SF (t (78) -3.790, *p* <.001, Cohen’s d= -0.850), and the AFQ-Y8 (t (78) 5.338, *p* <.001, d = 1.197). The SCS-SF scores of the control group were higher than ACTi. The AFQ-Y8 scores of the control group were lower than ACTi.

**Table 3:** Frequencies, means, and standard deviations (in parentheses) of the sociodemographic and measurements variables of the baseline and the p*ost-intervention* measurement

Variable	ACTi (*n* = 37)	Control Group (*n* = 43)	Statistics	
Demographics				
Sex (girls/boys)	28/9	28/15	χ2(1) = 1.056	
Age in years	16.865 (0.347)	17.023 (0.34)	*t* (78) -2.046*	
**Baseline measurements (ACT vs. Control Group)**				CI Lower; CI Upper
CDRS-SF	20.11 (6.354)	21.56 (6.566)	*t* (78) -1.000, *Cohen’s d=* -0.224	-4.338; 1.438
SCS-SF	28.189 (5.631)	34.442 (8.564)	*t* (78) -3.790***, *Cohen’s d=* -0.850	-9.537; -2.968
AFQ-Y8	21.676 (4.096)	14.930 (6.678)	*t* (78) 5.338***, *Cohen’s d =* 1.197	4.23; 9.261
**Post-intervention measurement (ACT vs. Control Group)**				CI Lower; CI Upper
CDRS-SF	24.00 (7.299)	20.91 (5.273)	*t* (78) -2.193**, *Cohen’s d* = 0.492	0.285; 5.901
SCS-SF	35.568 (3.731)	33.116 (6.939)	*t* (78) 1.922**, *Cohen’s d =* 0.431	0.088; 4.990
AFQ-Y8	18.460 (5.064)	15.791 (4.591)	*t* (78) 2.472**, *Cohen’s d =* 0.554	0.519; 4.818

Note: *ACTi: ACT intervention;* CI Lower: Confidence Interval Lower, CI Upper: Confidence Interval Upper; ***p <.001, **p <.01, *p <.05

The participants in the ACTi group scored significantly higher on the CD-RISC-SF (t(78) = -2.193, *p* <.01, d = 0.492) and the SCS-SF (t(78) = 1.922, *p* <.01, d = 0.431) compared to the control group. Conversely, the control group scored significantly lower on the AFQ-Y8 than the ACTi group (t(78) = 2.472, *p* <.01, d = 0.554), indicating reduced psychological inflexibility. These results suggest that the ACTi contributed to increased resilience and self-compassion compared to the control condition.indicating higher psychological inflexibility in the ACTi group. However, this result should be interpreted in light of baseline differences and the overall direction of change from baseline to post-intervention.

**Table 4:** Results of repeated measure of the baseline and the post-intervention measurement at the ACT and the control group

	Baseline	Post-intervention	Interaction group x time	
**ACTi (Baseline vs. Post-intervention)**				CI Lower; CI Upper
CDRS-SF	20.11 (6.354)	24.00 (7.299)	*t* (36) -3.431***, *Cohen’s d=* -0.564	-0.908; -0.213
SCS-SF	28.189 (5.631)	35.568 (3.731)	*t* (36) -7.031***, *Cohen’s d=* -1.156	-1.569; -0.733
AFQ-Y8	21.676 (4.096)	18.46 (5.064)	*t* (36) 3.414***, *Cohen’s d = 0*.561	0.211; 0.905
**Control Group (Baseline vs. Post-intervention)**				
CDRS-SF	21.56 (6.566)	20.91 (5.273)	*t* (42) -0.955, *Cohen’s d* = 0.492	-0.156; 0.445
SCS-SF	34.442 (8.564)	33.116 (6.939)	*t* (42) 1.066, *Cohen’s d =* 0.163	-0.139; 0.463
AFQ-Y8	14.93 (6.678)	15.791 (4.591)	*t* (42) -0.913, *Cohen’s d=* -0.139	-0.439; 0.162

Note: *ACTi: ACT intervention;* CI Lower: Confidence Interval Lower, CI Upper: Confidence Interval Upper; ***p <.001, **p <.01, *p <.05

The participant’s baseline and post-intervention in the control group did not differ in the CD-RISC-SF (t (42) -0.955, *p* >.05, Cohen’s d = 0.492), the SCS-SF (t (42) 1.066, *p* >.05, Cohen’s d = 0.163), and in the AFQ-Y8 (t (42) -0.913, *p* >.05, Cohen’s d= -0.139). There was no significant interaction effect for the results of baseline and post-intervention in the control Group.

**Fig. 2:**
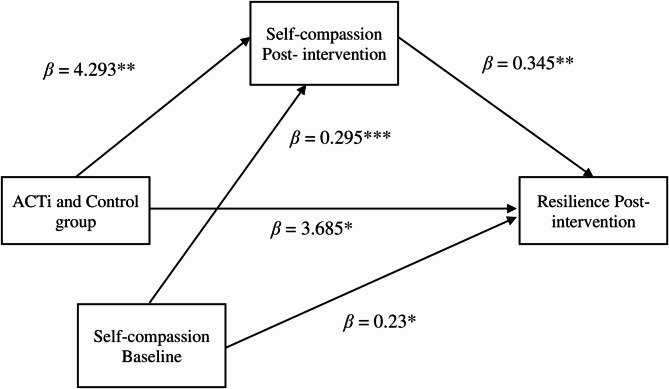
Mediation model in which psychological inflexibility post-intervention association levels mediate the relationships between ACTi and control group and resilience post- intervention. ***Note***: *ACTi: ACT intervention;* ***p <.001, **p <.01, *p <.05

In the first mediation model, ACTi and the control group were significantly related to the CD-RISC-SF (β = 3.685, *p* <.05, se = 1.456). ACTi and the control group predicted the SCS-SF post-intervention (β = 4.293, *p* <.01, se = 1.296). When the SCS-SF post-intervention was controlled via the SCS-SF Baseline, significantly influenced CD-RISC-SF (β = 0.345, *p* <.01, se = 0.12). The SCS-SF Baseline significantly influenced CD-RISC-SF (β = 0.23, *p* <.05, se = 0.093) (see Fig. 2).

As shown in Table 5, the unstandardized mediation effect of the SCS-SF post-intervention scores (β = 1.48 Boot CI [0.332, 3.06]) was obtained by multiplying a and b in the previous regression model. Since the mediation decision was made by having the lower and upper levels of the Bootstrap CI be on the same side of zero (0), the mediation effect was observed because the SCS-SF post-intervention scores were on the same (positive) side of the Bootstrap CI.

**Table 5:** The model coefficients in the Estimation of the CDRS-SF by means of the SCS-SF and AFQ-Y8 post-intervention as a result of the use of the SCS-SF and AFQ-Y8 baseline of the acti and the control group as covariance

X	M	U_1_	Y	Indirect Effect (a x b)	Boot SE	Indirect effect 9% CI
ACTi vs. Control Group	SCS-SF Post-intervention	SCS-SF Baseline	CDRS-SF Post-intervention	1.48	0.7	0.332, 3.06
	AFQ-Y8 Post-intervention	AFQ-Y8 Baseline	CDRS-SF Post-intervention	-0.205	0.491	-1.209, 0.823

Note: X: Independent variable ACTi and Control Group), M: Mediator (AFQ-Y8 Post-intervention, SCS-SF Post-intervention), U_1_: Covariance variable (SCS-SF Baseline, AFQ-Y8 Baseline); DV: Dependent variable (CDRS), Indirect effect: if the 95% CI did not include 0, it indicated that the indirect effect is significant

**Fig. 3:**
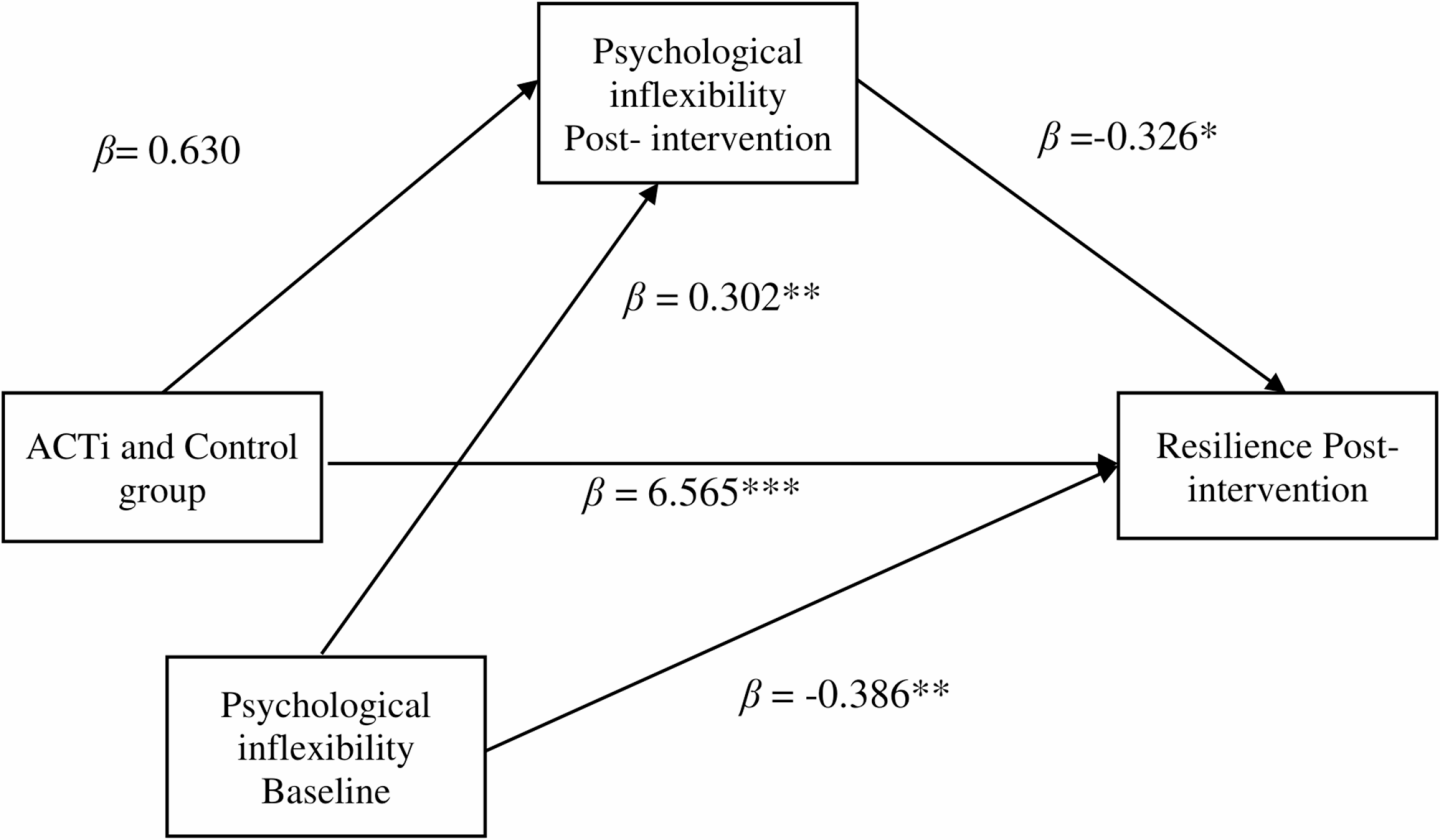
Mediation model in which psychological inflexibility post-intervention association levels mediate the relationships between ACTi and control group and resilience post-intervention. ***Note***: *ACTi: ACT intervention;* ***p <.001, **p <.01, *p <.05

In the second mediation model, ACTi and the control group were significant related to the CD-RISC-SF (β = 6.565, *p* <.001, se = 1.455). The ACTi and control group did not predict the AFQ-Y8 post-intervention (β = 0.629, se = 1.188). When the AFQ-Y8 post-intervention was controlled via the AFQ-Y8 Baseline, significantly influenced CD-RISC-SF (β = -0.326, *p* <.05, se = 0.139). The AFQ-Y8 Baseline significantly influenced CD-RISC-SF (β = -0.386, *p* <.01, se = 0.119) (see Fig. 3).

As shown in Table 5, the unstandardized mediation effect of AFQ-Y8 post-intervention scores (β = -0.205 Boot CI [-1.209, 0.823]) was obtained by multiplying a and b in the previous regression model. Since the mediation decision was made by having the lower and upper levels of the Bootstrap CI on the same side of zero (0), the mediation effect was not observed, because the AFQ-Y8 post-intervention scores were on different sides of the Bootstrap CI.

## Discussion

This study is one of the first to investigate the resilience of non-clinical adolescents with low socioeconomic status in Türkiye and one of the first studies to examine the effect of ACT on resilience. Previous studies have demonstrated that children with low socioeconomic status are more prone to mental health problems [53–55]. Alho et al. [56] found that peer mental disorder diagnoses during adolescence increase the risk of developing a mental disorder later in life. Therefore, understanding the mechanisms underlying protective properties against vulnerabilities is important to develop effective interventions to enhance their resilience.

Currently, only a few studies have supported the effectiveness of ACT in improving resilience among adolescents, but the evidence is limited because two of these studies are in Persian [57–60]. Thus, the current study fills a gap in the literature by comparing ACTi with a medium-sized sample (*N* = 80). The findings suggest that ACTi may have significant effects of medium magnitude over time. Additionally, the results indicate improvements in all three conditions—psychological flexibility, resilience, and self-compassion—following the intervention.

It was found that the resilience and self-compassion of the participants who participated in the ACTi increased, while their psychological inflexibility levels decreased. Conversely, there were no statistically significant changes in resilience, self-compassion, or psychological inflexibility in the control group that did not receive any intervention. These findings suggest that ACTi was associated with improvements in resilience and self-compassion, and reductions in psychological inflexibility. However, without an active control group, causal attribution remains tentative.

When self-compassion pretest scores were considered, the results revealed that self-compassion posttest scores predicted resilience posttest scores in both the intervention and control groups that completed the ACTi. When psychological inflexibility pretest scores were considered, psychological inflexibility posttest scores predicted resilience posttest scores of the intervention and control groups that completed the ACTi.

It is important to note that significant baseline differences were observed between the ACTi and control groups in terms of self-compassion and psychological inflexibility. These pre-existing differences may have impacted the observed intervention effects and should be interpreted cautiously. Future studies should consider statistical adjustments for baseline imbalances or employ stratified randomization techniques.

Furthermore, although self-compassion emerged as a significant mediator in the relationship between ACTi and resilience, psychological inflexibility did not show a similar effect. This may be due to the limited statistical power or the timing of post-intervention measurement. Additional studies are needed to clarify the temporal relationship and mechanisms between ACT processes and resilience outcomes.

Potential mental health issues in adolescents can have significant and long-lasting individual and societal effects. Therefore, it is essential to identify the psychological processes that help prevent or alleviate stress and related symptoms in adolescents, thereby increasing their likelihood of success in school. As a result, this study provides valuable evidence that positive interventions targeting individuals from low socioeconomic backgrounds may serve as protective factors.

Llistosella et al. [31] demonstrated through their meta-analysis that interventions involving social and emotional learning, counseling or mentoring, mindfulness, and social contact did not yield significant effects on resilience. These approaches alone were insufficient in enhancing resilience. In contrast, the present study implemented acceptance and commitment therapy to bolster resilience, while also incorporating techniques of mindfulness and self-compassion. The findings indicate that ACTi promotes resilience and that its effects are mediated by self-compassion. The results suggest that self-compassion partially mediates the relationship between ACTi and resilience, indicating that improvements in self-compassion may help explain the intervention’s impact on resilience. Additionally, psychological flexibility, which consists of components such as acceptance, moment-to-moment awareness, values, and high self-compassion, plays a significant role in this process.

### Limitations

In the current study has several limitations. First, the relatively small sample size (*N* = 80) restricts the generalizability and may increase the likelihood of Type II errors. Second, the ACTi and control groups showed significant baseline differences in self-compassion and psychological inflexibility, potentially influencing the outcomes independently of the intervention. Third, the absence of an active control group prevents a clear attribution of observed changes solely to ACT-based techniques, as non-specific factors such as participant expectation or group dynamics could have contributed.

Fourth, the study relied heavily on self-report measures, which are susceptible to response biases, including social desirability. Despite the use of validated instruments, future research should consider integrating objective outcome indicators such as teacher ratings, behavioral observations, or physiological measures. Lastly, this research focused exclusively on individual-level protective factors. Future studies should aim to explore contextual and systemic variables—such as school climate, peer support, and family resilience—when evaluating the impact of resilience-promoting interventions.

## Conclusions

The current research indicates that psychological flexibility is associated with enhanced resilience among adolescents, with self-compassion serving as a mediating factor. These findings are consistent with the view that psychological flexibility [36, 61] is associated with resilience and may play a role in protecting against psychological difficulties. Nonetheless, further investigations are necessary to explore the characteristics of the adolescent population represented in this study, their developmental trajectories, and the causal relationships involving depth of change as well as individual and contextual influences on well-being. These results suggest that brief interventions targeting psychological flexibility may hold promise for supporting adolescent well-being, particularly among those experiencing distress. However, further replication is needed across diverse populations and settings.

## Data Availability

Data are available in a public, open-access repository (Mendeley Data, V2, doi: 10.17632/zw366xjg6h.2).
